# Supplementary material for: Variable orthogonality of serine integrase interactions within the ϕC31 family
Source: Sci Rep. 2024 Nov 1;14:26280. doi: 10.1038/s41598-024-77570-9 (PMC11530663; doi:10.1038/s41598-024-77570-9)
Supplement: Supplementary file 1 — Supplementary Material 1 [file 41598_2024_77570_MOESM1_ESM.pdf]

A) ϕC31-Int-RDF fusions

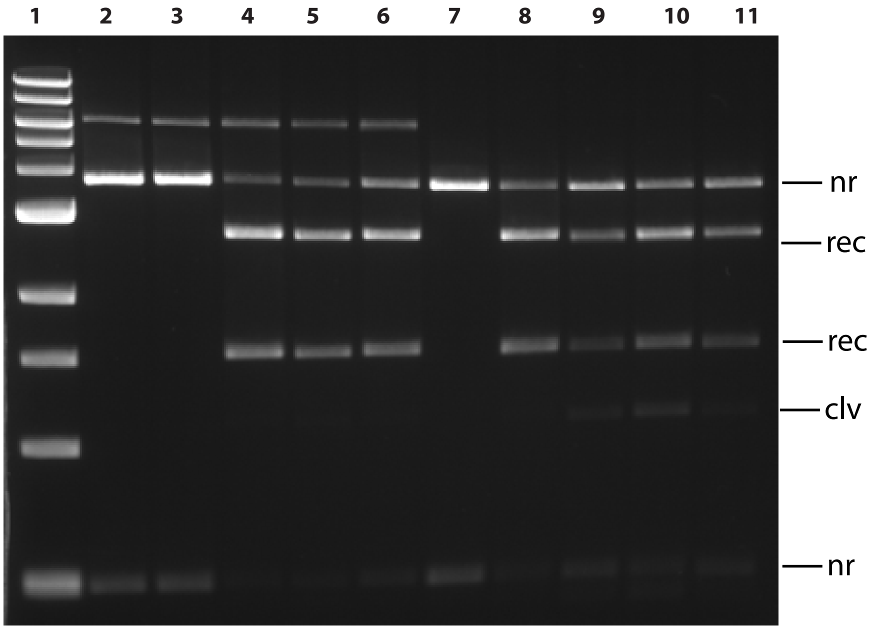

1. 1kb ladder

attR x attL

2. PDB
3. ϕC31-Int
4. ϕC31-Int-ϕC31-RDF Fusion
5. ϕC31-Int-ϕBT1-RDF Fusion
6. ϕC31-Int-TG1-RDF Fusion

attP x attB

7. PDB
8. ϕC31-Int
9. ϕC31-Int-ϕC31-RDF Fusion
10. ϕC31-Int-ϕBT1-RDF Fusion
11. ϕC31-Int-TG1-RDF Fusion

B) ϕC31-Int and RDFs as separate proteins

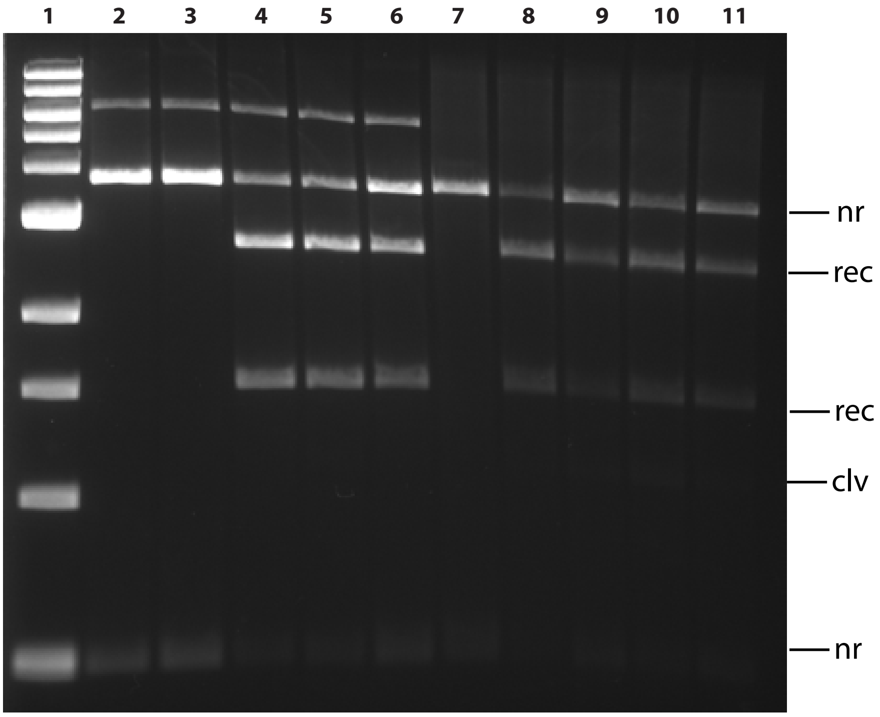

1. 1kb ladder

attR x attL

2. PDB
3. ϕC31-Int
4. ϕC31-Int + ϕC31-RDF
5. ϕC31-Int + ϕBT1-RDF
6. ϕC31-Int + TG1-RDF

attP x attB

7. PDB
8. ϕC31-Int
9. ϕC31-Int + ϕC31-RDF
10. ϕC31-Int + ϕBT1-RDF
11. ϕC31-Int + TG1-RDF

Figure S1: In vitro recombination reactions of ϕC31 integrase

A) ϕBT1-Int-Fusion_RL_PB

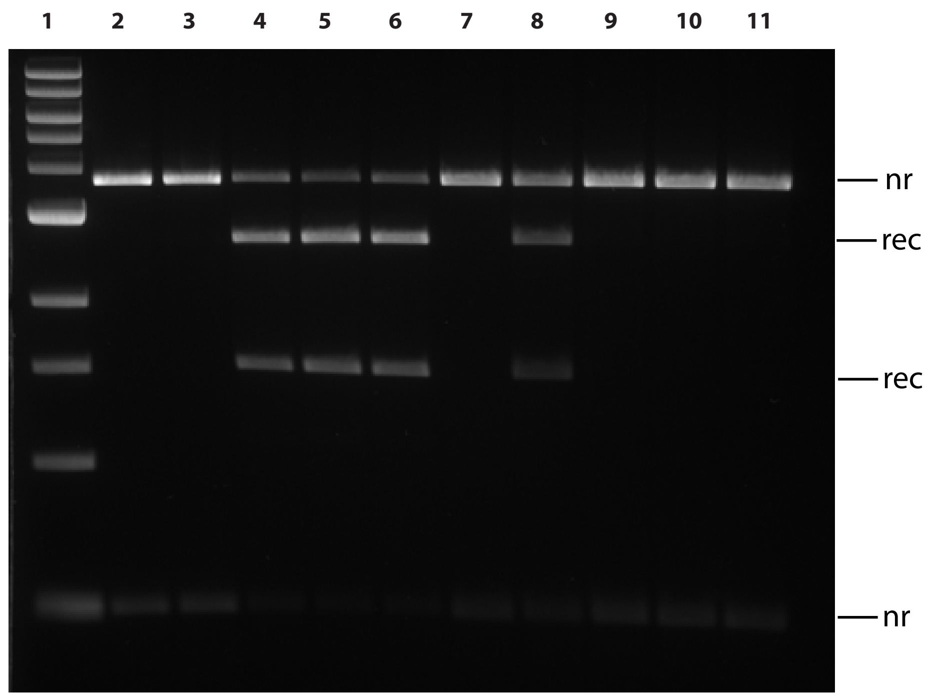

1. 1kb ladder

attR x attL

2. PDB
3. ϕBT1-Int
4. ϕBT1-Int-ϕC31-RDF Fusion
5. ϕBT1-Int-ϕBT1-RDF Fusion
6. ϕBT1-Int-TG1-RDF Fusion

attP x attB

7. PDB
8. ϕBT1-Int
9. ϕBT1-Int-ϕC31-RDF Fusion
10. ϕBT1-Int-ϕBT1-RDF Fusion
11. ϕBT1-Int-TG1-RDF Fusion

B) ϕBT1-Int and RDFs as separate proteins

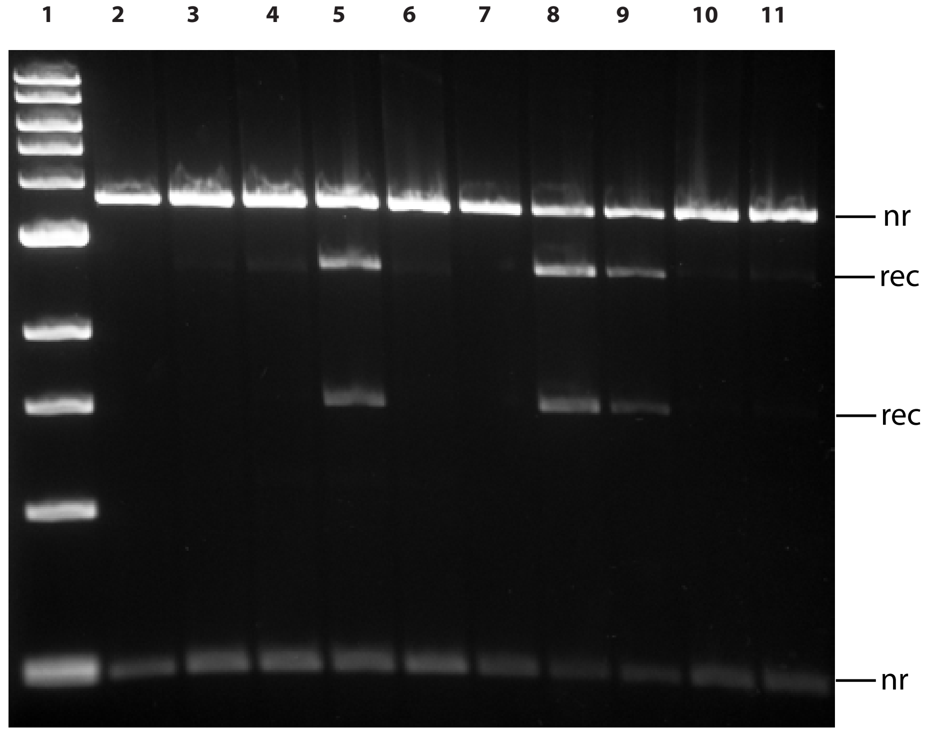

1. 1kb ladder

attR x attL

2. PDB
3. ϕBT1-Int
4. ϕBT1-Int + ϕC31-RDF
5. ϕBT1-Int + ϕBT1-RDF
6. ϕBT1-Int + TG1-RDF

attP x attB

7. PDB
8. ϕBT1-Int
9. ϕBT1-Int + ϕC31-RDF
10. ϕBT1-Int + ϕBT1-RDF
11. ϕBT1-Int + TG1-RDF

Figure S2: In vitro recombination reactions of ϕBT1 integrase

A) TG1-Int-RDF fusions

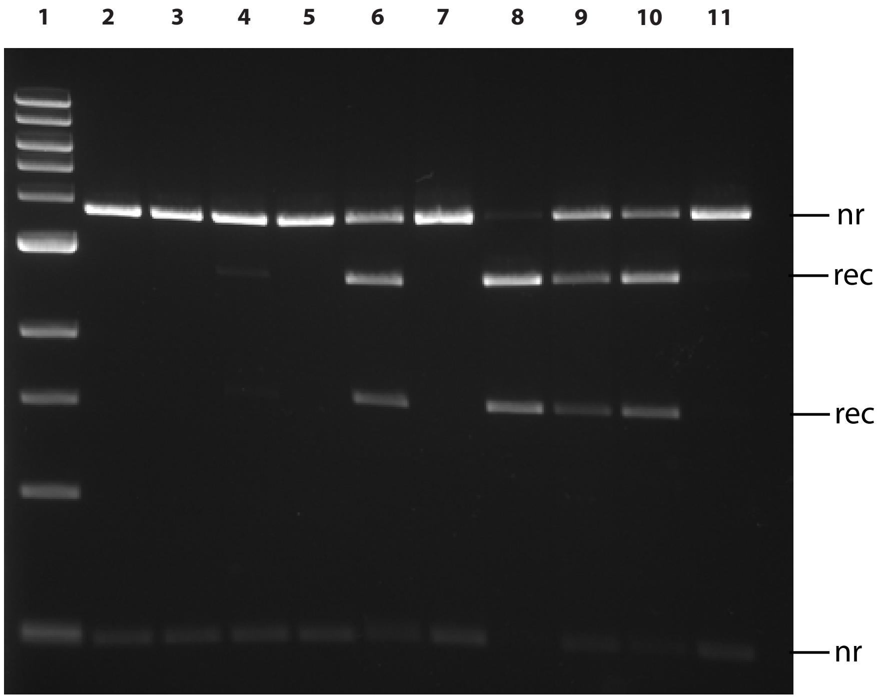

1. 1kb ladder

attR x attL

2. PDB
3. TG1-Int
4. TG1-Int-ϕC31-RDF Fusion
5. TG1-Int-ϕBT1-RDF Fusion
6. TG1-Int-TG1-RDF Fusion

attP x attB

7. PDB
8. TG1-Int
9. TG1-Int-ϕC31-RDF Fusion
10. TG1-Int-ϕBT1-RDF Fusion
11. TG1-Int-TG1-RDF Fusion

B) TG1-Int and RDFs as separate proteins

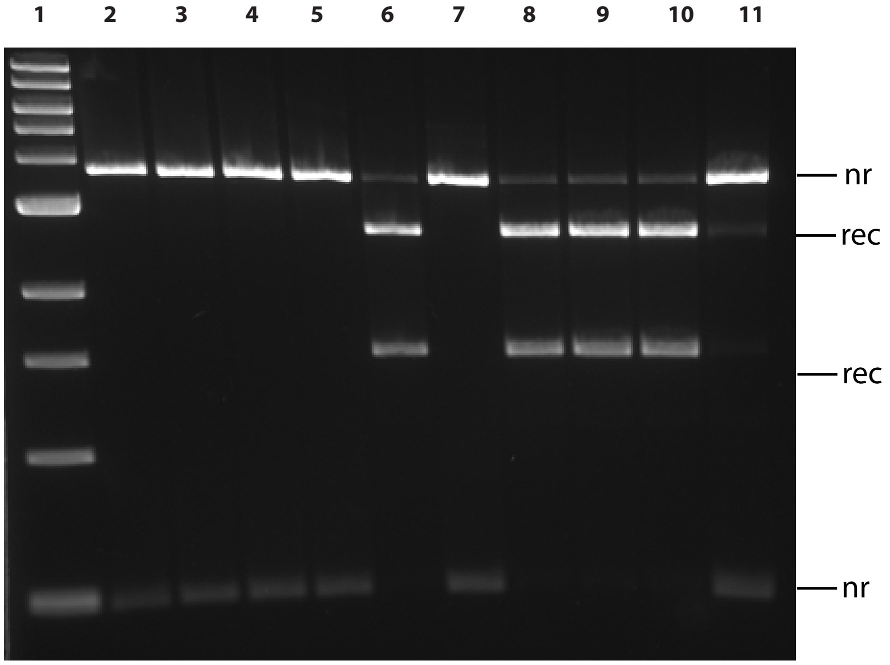

1. 1kb ladder

attR x attL

2. PDB
3. TG1-Int
4. TG1-Int + ϕC31-RDF
5. TG1-Int + ϕBT1-RDF
6. TG1-Int + TG1-RDF

attP x attB

7. PDB
8. TG1-Int
9. TG1-Int + ϕC31-RDF
10. TG1-Int + ϕBT1-RDF
11. TG1-Int + TG1-RDF

Figure S3: In vitro recombination reactions of TG1 integrase
